# Reliability of a Scale for the Evaluation of Generativity Relative to Health (GeReH) in the Mexican Population in the Aging Process

**DOI:** 10.3390/diagnostics11101833

**Published:** 2021-10-03

**Authors:** Otilia Aurora Ramírez-Arellano, Mirna García-Méndez, Juan Garduño-Espinosa, Luis Alberto Vargas-Guadarrama, Víctor Manuel Mendoza-Núñez

**Affiliations:** 1 Unidad de Investigación en Gerontología, Facultad de Estudios Superiores Zaragoza, Universidad Nacional Autónoma de México, Ciudad de México 09230, Mexico; otiliaauroraramirezarellano@gmail.com; 2 Carrera de Psicología, Facultad de Estudios Superiores Zaragoza, Universidad Nacional Autónoma de México, Ciudad de México 09230, Mexico; mina@unam.mx; 3 Hospital Infantil de Mexico “Federico Gómez”, Secretaría de Salud, Ciudad de México 06720, Mexico; juan.gardunoe@gmail.com; 4 Instituto de Investigaciones Antropológicas, Universidad Nacional Autónoma de México, Ciudad de México 04510, Mexico; vargas.luisalberto@gmail.com

**Keywords:** GeReH, Mexican older adults, generativity, healthy aging, telecare, virtual social networks

## Abstract

Generativity is a quality that allows the person to do something for others. In teaching, caring for grandchildren, or volunteering, the generative person contributes to the people around him and at the same time must maintain self-care for good health and functionality. In this sense, an individual in good health has the potential to contribute to the well-being of others. Likewise, with adequate self-esteem, the generative person can love himself, take care of himself and others; in this affective representation, satisfaction can be perceived from the recognition that others make of his transmission of experiences. The most used scales that measure generativity in the gerontological field are the Loyola Generativity Scale (LGS) and the Generative Behavior Control List (GBC). However, they do not recognize generative health-related behavior. The purpose of this study was to design a scale to assess generativity relative to health (GeReH) and analyze its psychometric properties in an aging population (45 years and over) in Mexico, considering its internal structure, reliability, and relationship with self-esteem. Method: A non-experimental cross-sectional study was carried out with a single group considering three stages: (i) design of the GeReH scale and (ii) psychometric properties of the GeReH scale, and (iii) the GeReH’s relationship with self-esteem. This type of instrument will allow identifying the profile of people willing to be independent and support their peers, considering the use of technological devices for community telecare, such as smartphones and personal computers, through the use of social networks such as “Facebook”, “WhatsApp”, and “Zoom” among others, is essential, especially since more and more older adults are users of these devices and virtual community social networks. The participants were 450 adults aged 45 years and over, of whom 296 women and 154 men; 235 people lived in rural areas and 215 people in urban areas. Inclusion criteria: independent in basic and instrumental activities of daily living. Exclusion criteria: cognitive alterations, no training or work history in health care. In the first stage, the GeReH design was carried out divided into three phases: i) elaboration of 258 items by a group of researchers in accordance with the proposed construct for the instrument, ii) focus group to know the meanings of adults regarding the items, iii) expert consultation for item analysis, which resulted in 90 items. In the second stage, the psychometric properties of the scale were analyzed, proceeding to the statistical analysis. Results: Bias, kurtosis, and total item correlation were analyzed, eliminating 17 items. KMO 0.904 values and Bartlett’s test of sphericity (X^2^ = 2717, gl = 190, *p* < 0.0001) were obtained. In the third stage, the correlation of the GeReH score with the Self-Esteem Inventory was determined. Orthogonal rotation (Oblimin) was used, obtaining a total explained variance of the generativity construct of 44.2% with a global Omega McDonald reliability coefficient of 0.887, which yielded five factors: F1 = Generative attitude; F2 = Satisfaction; F3 = Volunteering; F4 = Support Networks; F5 = Social support offered. In this sense, the design of a GeReH of 20 items with psychometric properties. Correspondingly, significant positive correlations were observed between the GeReH score and the Self-Esteem Inventory, especially in factor 2 relative to satisfaction. Conclusion: GeReH is an instrument with reliable psychometric properties that could be applied in populations with similar characteristics. In addition to considering the use of technological devices, for the optimal use of media and social networks, such as “Facebook”, “WhatsApp”, “E-mail”, and “Zoom”, among others.

## 1. Introduction

Generativity is a quality that allows an individual to carry out a task that benefits the people around him or to carry out activities that contribute to the improvement of the environment in which he lives. It is a construct that falls within the theory of psychosocial development, based on the notion that each person is constituted and transformed in a convergence between the bio-psycho-social [1].

Erikson pointed out that being generative is something that can be achieved in the penultimate stage of life (40–60 years) when the person shows interest, has goals, and performs activities in which he transmits something to others or cares for others [1].

Thus, some ways in which generativity makes it possible to leave a mark on the world are: to offer help, serve, contribute, share or contribute, which leads the generative person to meet positively oriented goals that allow him to excel [2]. Being generative can be a source of multiple benefits because it leaves a positive mark on the lives of others and offers satisfaction. The generative person can then take care of others, create things or carry out activities that make the world a better place to live; examples of these are teaching, caring for grandchildren, volunteering, and participating in community or institutional programs [3].

It is convenient to emphasize that generativity can be divided into two phases. First, there must be generative interest, and then to carry out generative behavior, that is, there must be the cognitive and affective component that motivates an intention for the common good, which can be concretized in generative action [4]. In the second phase, the person carries out behaviors focused on others; these are specific activities for the well-being of others that offer satisfaction [5,6].

Essentially, maintaining physical and cognitive capacities allows the generative person to use their self-care strategies for health, learn new knowledge related to health care and share it with others. Thus, breaking one of the great myths about aging, the idea that old age is too late to adopt and/or maintain healthy habits [7].

Hence, the behavioral manifestations of generativity are related to better well-being and better physical health, reduced disability, and lower mortality [8,9]. Certainly, in the area of mental health, generative acts can repair suffering, becoming important in the restoration of oneself, which means that the person perceives the future as something promising [10].

Likewise, there is a positive effect of self-esteem with satisfaction with life in old age; satisfaction can be manifested in the form of recognition, respect is an example since if the generative person feels respected, their well-being can increase [11,12]; therefore, satisfaction advances hand in hand with good self-esteem that allows us to identify positive aspects of aging, its acceptance and the breakdown of prejudices.

The contexts in which people develop, the interactions they carry out, as well as their intrinsic capacities and functional capacity, allow them to be and do what is important for them during their aging process. In this sense, the World Health Organization (2015) has pointed out these characteristics as key elements of healthy aging, defined as “the process of promoting and maintaining the functional capacity that allows well-being into old age” [13].

Within the framework of human development, it has been proposed that aging begins in the fifth decade of life, around the age of 45, since at this stage there are patent biological, physical, psychological, and social changes related to aging in the majority of the population [14]. This approach is more inclusive and prospective than the one proposed by the WHO, which limits it to old age.

Gruenewald et al. (2012) refer that generativity is associated with more favorable trajectories of physical functioning and longevity in older adults [8]. Likewise, Arias and Iglesias-Parro (2015) point out that being generative has a significant effect on both objective and subjective health, particularly in activities that are relevant for older people where they guide their adaptive efforts to achieve the desired states [15].

In any case, health and generativity are part of the same cycle since being generative is necessary to be functional and have good health, which allows making use of the ability to freely undertake whatever is decided. Something important to note is that, in addition to having health and the potential to be generative, there must be an environment that allows the development of generative projects [16]. In this regard, the cultural context plays a crucial role in maintaining or creating spaces to be generative; some cultures favor and educate prosocial behavior more than others [17].

Over time, generativity has been evaluated with various instruments, for example: (i) qualification of the stages of adult life, (ii) Eriksonian measure of psychosocial development, (iii) assessment of personal plans for the future, (iv) self-report questionnaire to assess Erikson’s stages modified version of Erikson’s inventory of psychosocial stages, “Psychosocial Development Balance Inventory”, (v) satisfactory human development perception test (vi), generativity measurement of parents, and (vii) assessment of the interests and attitudes of adults [18].

Likewise, in the gerontological field, the most widely used scales to measure generativity are the Loyola Generativity Scale (LGS) [5,19], which has been adapted for different populations [18] and the Generative Behavior Control List (GBC) [20], in which participation in personal, family and community productive projects is valued above all, it should be clarified that these instruments do not consider generativity related to health.

In this context, we propose that it is necessary to have an instrument that allows assessing whether the evaluated person has an interest and is aware of the generative value of self-care for health, to be shared with others, in order to fulfill their other projects of a personal and social nature. Such information could be of great use to establish community training programs for people in the aging process for the development and implementation of generative strategies for health.

Generative experiences are anchored in a social ecology of the individual, revealing a psychosocial space that connects the person with their social environment, which is necessary to have generative achievements [21], thereby enhancing human and social capital, social support networks, and mutual trust within the framework of society, which facilitates cooperative action between citizens and institutions [22].

Some studies have reported health and well-being indices related to people’s social capital, determined by social support networks with human capital that positively influences lifestyles and self-care for health [23,24]. In this sense, the perspective of human and social capital must be incorporated in the promotion of public health, with a focus on social determinants [25,26].

Generativity is gestated from a cognitive element that is interest and is linked to one or more effective elements that can be satisfaction, self-esteem, or motivation, which implies that the person can assume a generative goal because it emotionally rewards them something positive, a crucial element is a behavior since an individual can carry out generative activities in different areas aimed at different social actors: family, friends, community or institutions with which social bonds are generated that allow building a bridge between the person and society if this goes from being an isolated behavior to a lifestyle, it means that habits have been created that allow the cycle of interpersonal relationships necessary to be generative to continue [4,5,6].

The level of self-esteem of the individual is related to the perception of himself compared to his personal values. These fundamental values are developed through the process of socialization, to the extent that the distance between the ideal self and the real self is small, self-esteem is higher. On the contrary, the greater the distance, the lower the self-esteem, even when the person is viewed positively by others [27]. It is adulthood, the time when the individual reaches the most complete physical, psychological, and social maturity, a moment in which the possible relationship between self-esteem and generativity can be identified.

Aging as part of human development is the period of the human life cycle par excellence to be generative, so self-care behavior for health is a key element to be well and thus have better conditions to be functional and proactive. The empowerment and use of social support networks become relevant as strategies to know what to transmit. In this framework, the construct of generativity related to health is proposed, establishing health self-care as a key element, considering empowerment and the use of social support networks as strategies to maintain physical and mental functionality, and consequently, independence and autonomy during aging. For this reason, the following are incorporated as fundamental components of the structure: (i) generative attitude, (ii) satisfaction with the implemented activities, (iii) willingness to support others as volunteers, (iv) recognize and use social networks support and (v) recognize the person’s ability to help others [2,6,7,28].

Agentic generativity becomes relevant since it implies the extension of the self through leadership, productive, scientific, or business activity. Within this framework, training in the use of technological devices for community telecare, such as smartphones and personal computers, through the use of communication media and virtual social networks such as “Facebook”, “WhatsApp”, “Zoom”, and “E-mail“, among others, is fundamental, especially since more and more older adults are users of these devices, media and virtual social networks [29].

COVID-19 is an example of the relevance and need for the use of Internet-enabled smart devices for remote monitoring through body sensors for medical care and the participation of patients online in prevention, detection, and treatment [30,31,32]. In this regard, the current situation deserves to recognize the human and social capital that people in the aging process (≥45 years) represent for their own care and that of others, considering a current community approach that integrates empowerment and optimizing the use of available technology, to achieve maximum health and well-being during aging with the perspective of generativity relative to health.

For this reason, the purpose of this study was to design a scale to assess health-related generativity and analyze its psychometric properties in a population aged 45 years and over in Mexico, considering its internal structure, reliability, and relationship with self-esteem.

## 2. Materials and Method

A non-experimental cross-sectional study was carried out with a single group [33], considering three stages: (i) design of the GeReH scale and (ii) psychometric properties of the GeReH scale, (iii) relationship of generativity with self-esteem. The participants were 450 adults aged 45 years and over, of whom 296 women and 154 men; 235 people lived in rural areas and 215 people in urban areas. Inclusion criteria: independent in basic and instrumental activities of daily living. Exclusion criteria: cognitive alterations, no training or work history in health care.

All the instruments used in the study were digitized using the application *Google Forms* with a standardized protocol to guarantee their reliability.

### 2.1. Screening Instruments

#### 2.1.1. Basic Activities of Daily Living (BADL)

The BADL were evaluated through the Katz Index, in which 6 physical capacities are considered: bathing, dressing, moving from bed, eating, personal grooming, and walking in the room. It has a structured format of 6 points, each one with two answer options: independent = 2 and dependent = 1. The minimum score is 6, and the maximum is 12 [34]. All participants had 12 points.

#### 2.1.2. Instrumental Activities of Daily Living (IADL)

The IADL were evaluated with the Lawton and Brody Functionality Scale. It is a scale of seven activities: using the telephone and transportation, shopping, preparing food, doing household chores, managing their medication and money. Each one considers three response options: independent with a value of 3, with assistance with a value of 2, and dependent with a value of 1. The minimum score is 7, and the maximum is 21 [35]. All the participants had 21 points.

#### 2.1.3. Cognitive Function (CF)

CF was evaluated through the application of the Folstein Minimental Scale, validated for the Mexican population. The scale is made up of 30 questions integrated into 5 sections: orientation, registration, attention and calculation, language, and deferred memory. In all cases, the correct responses of the person are scored with the number 1; when they are incorrect, the value is zero, the maximum score is 30, a score ≤23 was considered as the cut-off point for cognitive impairment [36].

#### 2.1.4. Sociodemographic Data Questionnaire (SDQ)

An SDQ was used to record data on age, sex, marital status, place of residence, schooling, health status, and participation in a community group.

### 2.2. GeReH Design

#### 2.2.1. Elaboration of Items

The GeReH scale was made up of a database of 258 items on generativity and health proposed by a group of 6 researchers (2 gerontologists, 2 psychologists, and 2 nurses) from the Gerontology Research Unit at the National Autonomous University of Mexico (FES Zaragoza). The items were prepared based on the theoretical notions of McAdams et al. (2004) that include the components: internal desires, cultural demand, generative interest, commitment, belief, generative action, and narration [37], considering the elements of the proposed construct on generativity relative to health.

#### 2.2.2. Focus Group

In order to reinforce the items on the scale, a focus group was organized in order to obtain information on the research topic [38]. Nine elderly people from the community collaborated, seven women between 48 and 70 years old and two men between 52 and 53 years old, respectively; two of them with basic schooling, four with a technical level, and three with a higher level. In terms of occupation, they were housewives, retirees, an accountant, and a teacher.

#### 2.2.3. Expert Consultation

Three researchers from the National Autonomous University of Mexico with experience in instrument design and in the subject of aging were invited in order to analyze the items according to their relevance, relevance, and sufficiency in the dimensions: cultural demand, internal desires, generative interest, commitment, belief and generative action [9].

#### 2.2.4. Construct Components for the GeReH Design

The construct analysis allowed identifying the following components:

(i) Generative attitude considers the thoughts, affections, and behaviors a generative person has and that are aimed at the well-being of the people around him, either through activities aimed at those people or because it contributes to the improvement of the environment in which they live. (ii) Satisfaction, feeling of well-being or pleasure that one has when a desire has been fulfilled, or a need has been covered. (iii) Volunteering, a series of activities (work) that by their own free decision the people who serve the community carry out. It can also be manifested in the contribution to improving the environment, which means that part of the time is dedicated to solidarity action without expecting any retribution for it. (iv) Support networks, ties that people establish among each other to help.

#### 2.2.5. Procedure

For the focus groups, those who had been part of the healthy aging program groups were invited. At the beginning of the focus group, the objective was read, and the participants were asked for their consent to record. Once they accepted, the guide was used to ask the planned questions. The session was recorded in audio. The duration was 2 h 4 min.

### 2.3. GeReH Psychometric Properties

With the results of the scale design, the psychometric properties of the GeReH were obtained according to the methodology established by Muñiz (2018) [39].

#### 2.3.1. Participants

A non-probabilistic convenience sampling was used, in which 450 people in the aging process participated voluntarily, 235 from the rural area (Oaxaca, Mexico), and 215 from the urban and suburban area of Mexico City. The age range was from 45 to 95 years (M = 57.5 SD ± 10.8), 65.8% women and 34.2% men. The sociodemographic characteristics of the participants are shown in Table 1., based on the MacCallum method, to determine the sample size for the factor analysis. The recommendation is a minimum of five participants per item for the psychometric evaluation of a measure, a criterion that was met in this study [40].

#### 2.3.2. Procedure

The application of the GeReH was carried out in two modalities with prior informed consent: (i) in person in the State of Oaxaca, Mexico, at the “Museo Casa del Pueblo de San Andrés Dinicuiti” and at the “Agencies of Santa María Tutla and Santiago del Río”. Five interviewers were trained for the application under the supervision of a researcher. In the cases of illiterate or disabled participants, they were supported by reading each of the items slowly aloud so that they could choose one of the five response options; (ii) digitalized online format, it was used for the population of the State of Mexico, due to the critical health situation of the country because of the country due to the SARS-CoV-2 pandemic. The format consisted of a digital version of the scale for which the Google Forms platform was used. In both versions, an agreement was confirmed, and a protocol was used to ensure the understanding of the items. The data were collected during June and July 2020.

### 2.4. Relationship between GeReH and Self-Esteem

The same sample and procedure were used for the psychometric properties of the GeReH scale of the present study to determine the relationship between GeReH and self-esteem.

#### 2.4.1. Self-Esteem

To determine self-esteem, the Rosenberg Self-Esteem Inventory (RSI) was used. It is a valid instrument for the Mexican population. It has an explained variance of 50.29% and a Cronbach’s alpha coefficient of 0.74 [41]. It consists of 10 items with Likert-type response options: (4) totally agree, (3) agree, (2) disagree, (1) totally disagree. The following scores are considered as a probable diagnosis: 30 to 40 points for high self-esteem, 26 to 29 points for medium self-esteem, and 25 points or less for low self-esteem.

#### 2.4.2. Relationship between Generativity and Self-Esteem

In order to explore the relationship between the generativity construct and self-esteem, the following hypothesis was generated: generativity is positively related to self-esteem given that an individual who cares for others has to perceive himself with qualities and abilities, proud of himself, useful, with success and positive attitude.

### 2.5. Statistical Analysis

The analysis of the items was carried out with the following statistics:

(a) Frequency distribution to obtain the percentage of response to each option of the items, the bias, and the kurtosis of every item;
(b) Student’s t-test for extreme groups in order to obtain the discrimination power of each item;
(c) Total item correlation corrected to identify inter-item correlation. The cut-off point for including the items in the analysis was 0.3 [42];
(d) Kaiser-Mayer-Olkin (KMO) normality analysis and Bartlett’s Sphericity Test in order to identify the feasibility of applying an exploratory factor analysis [43];
(e) Exploratory factor analysis (EFA) to determine the number and composition of the factors [44];
(f) McDonald’s Omega with the purpose of obtaining the reliability indices of the scale factors and the global scale. A value of 0.7 or higher indicates sufficient internal consistency for a new scale [45];
(g) Pearson’s bivariate correlation analysis between the GeReH and RSI factors.

The six mentioned procedures were carried out using the IBM/SPSS version 25.0 software.

## 3. Results

### 3.1. GeReH Scale Design

For the focus group, the information obtained was compiled in the Atlas ti 8.4 version 8.4 program to classify the *a priori* codes corresponding to the following dimensions: individual and social resources, interest, commitment, generative behavior, and social identity. *A posteriori,* the codes were elaborated: illness, self-care, relationship with young people, satisfaction, and networks. When labeling the audio transcript, 69 citations were obtained, from which semantic networks were detached that showed high concordance of the meanings expressed by the participants of the focus group with the items of the proposed instrument to evaluate generativity.

On the other hand, the experts modified the wording of several items. They indicated that double negation should be avoided in some, suggested eliminating similar items, omitting those that were not relevant, and requested to change the dimension of others, considering that they evaluated elements of another dimension.

The result of stage 1, the GeReH scale, was obtained, composed of 90 items with five response intervals: (1) never, (2) almost never, (3) sometimes, (4) almost always, (5) always. This version was validated by the consensus of experts in order to detect generative interest, considering the behavior, satisfaction, commitment, and goals of people within the framework of their own health and empathy for the care of others (Appendix A).

### 3.2. Psychometric Properties

For each item, all response options were chosen by a percentage of participants. No response options were obtained with values of zero, and the highest percentage of them was 76.7%. The bias values were less than ±1.5, which is why it is concluded that they are not drastically separated from normality. Three items had scores above ± 1.5, 66 with a bias of −2.36 and a kurtosis of 5.52 that was eliminated, items 68 and 84 had a bias of −1.68 and −1.72 with kurtosis of 2.50 and 2.54, respectively. Given the theoretical contribution of these two items to the construct, it was decided to keep them and observe their behavior in subsequent analyzes. The kurtosis values ranged from −0.07 to −2.54, except for item 66, which was eliminated. Total item correlations ranged from 0.05 to 0.66. As a result of this analysis, 17 items were eliminated.

With the remaining 73 items, the values of the Kaiser-Meyer-Olkin (KMO) = 0.904 sample adequacy measure and Bartlett’s sphericity test (X^2^ = 2717, gl = 190 *p* < 0.0001) were obtained. Based on this evidence, it is assumed that the data are sufficiently related to be subjected to the exploratory factor analysis (EFA) [44].

To obtain the factorial structure of the scale, an exploratory factor analysis (EFA) was performed using the unweighted least-squares extraction method. This extraction method was used to minimize the sum of squares of the differences between the observed and reproduced correlation matrices and to avoid saturations greater than unity and negative error variances factors [44]. Orthogonal rotation (Oblimin) was used. The criterion followed to consider an item as part of a factor was that it had a factorial weight of ≥0.40 and that it did not share factorial weight in another factor. These values indicate the degree of correspondence between the item and the factor. High loads indicate that the item is representative of the factor. So, it is desirable that each one has weight in only one factor, and values of 0.4 are considered reasonable [46]. Subsequently, the reliability index of each factor and the global scale was obtained through the McDonald’s Omega reliability coefficient. With this result of the analysis, the scale was made up of 20 items distributed in five factors. The total explained variance of the generativity construct was 44.2%, with a McDonald’s Omega global reliability coefficient of 0.877 (Table 2).

In Table 3, the results of the correlations of the generativity factors are shown. In this sense, the correlations between the items were positive, between low and moderate. This result was as expected since the items are conceptually interrelated. On the other hand, the total correlation between the corrected items and the results of the Student’s t-test for extreme groups is shown in Table 4.

### 3.3. Relationship between the GeReH and Self-Esteem

A Pearson bivariate correlation analysis was carried out between the GeReH factor score with the RSI. The results show statistically significant positive correlations between the GeReH and RSI scores, especially in the satisfaction factor (Table 5).

The GeReH scale, made up of 20 items organized into five factors, was originally constructed in Spanish (Appendix B). It is proposed in English in the same way (Appendix C).

## 4. Discussion

The present research aimed to design a scale to assess health-related generativity (GeReH) and analyze its psychometric properties in an aging population in Mexico, considering its internal structure, reliability, and relationship with self-esteem.

Regarding the scale design, a first validity check started from the writing of the items, for which it was key that a group of knowledgeable people with experience in the construction of measurement instruments evaluated and offered suggestions to improve it. In this regard, somehow, since the items were clear and the instrument was balanced and proportional to measure the generativity related to health in the aging process, it is also important to point out that the analysis of information provided by the focus group was essential for the detection and correction of errors in the meaning of the items. This, as a whole, was essential to have the 90-item version of the GeReH scale.

Although the 90-item version applied to 450 people from two states of Mexico did not show psychometric reliability in its entirety, this version could be useful as a screening questionnaire, considering that it is the result of validity by consensus of experts.

A scale must reflect a construct so that a large number of items become a reduced number called factors or dimensions without significant loss of information from the original items [47]. In this sense, the GeReH of 20 items complies with this rule from the analysis of its psychometric properties, so that the factorial structure obtained was made up of five factors, which have positive correlations with each other, which indicates probable independence between the factors that make up the construct of generativity.

GeReH factors are: (i) generative attitude, (ii) satisfaction, (iii) volunteering, (iv) support networks, (v) social support offered, thus showing the multidimensional nature of the concept of generativity related to health, in congruence with the proposed construct. Thereunder, the instrument includes the cognitive, affective, and behavioral elements that allow the individual to link up with the people around him to be generative in the context of health. Regarding the internal consistency values, acceptable McDonald’s Omega results were obtained both for the total scale and for the factors that comprise it. So, having a high score on all five factors means more generativity relative to health.

The exploratory factor analysis made it possible to have a short and easy-to-use scale where factors 1 and 2 were made up of five items each, factor 3 made up of four items, and factors 4 and 5 by three items each. Therefore, that there was no excess (over retention) nor a low number of items (support) for the GeReH factors, which is positive since the reproducibility of a factor is inversely proportional to the number of items that compose it [47]. In the GeReH, each factor selected met the criterion of having between three and five items [47,48,49], thus showing theoretical solidity and stability [48].

Often, the first factor on a scale is the one that shows the highest eigenvalue and, consequently, explains the largest percentage of the variance of the solution, collecting the essential characteristic of the construct under study [50]. In the case of the GeReH, the first two factors (with five items each) explain most of the variance and offer the highest reliability. The theoretical elements that stand out are: interest, behavior, and generative satisfaction, fundamental in the construct literature and taken from MacAdams and St Aubin (1993), Erikson (2000) [1,9], within the framework that behavior and generative concern are the most investigated aspects when it comes to the benefits of being generative, they provide satisfaction, desire to live and willingness to participate in social and community life linking the individual and the social in adulthood and beyond, that is, in the aging process.

The difficulty of measuring generativity is that it is a complex construction that must consider two levels: (i) individual, which implies cognition and affect, and (ii) psychosocial functioning, elements that must be evaluated simultaneously since they converge and diverge. In this sense, the inseparable and integral relationship between the individual and the collective, each forming and being formed by the other, is a key element, the generativity from which the richness of its measurement derives [51].

GeReH was made up of a first factor, called “generative attitude”, grouped items associated with generative interest, generative behavior, and positive perception for offering support. McAdams and Logan (2004) distinguish generative interest from generative behavior. The first has been defined as a favorable attitude toward generative issues, and the second as the performance of behaviors that represent a contribution and a benefit for the following generations [37].

Thus, generative interest refers to the cognitive and affective component that motivates an intention for the common good, which can be specified in behaviors focused on others and specific activities for the well-being of others that offer satisfaction to the generative person [5,6].

It should be noted that, after studying the differences between interest and generative behavior, Cheng (2009) attributed to the opportunities that people may have to put this generative interest into practice, based on variables such as sex, educational level, or marital status [5].

The second factor, “satisfaction”, grouped items related to the manifestation of positive emotions, optimism, achievement of goals, and good humor. Rubisntein et al. (2015) point out that satisfaction is something affirmative that can be manifested in the form of recognition [11], and Tabuchi et al. (2015) exemplify that when a person is respected for being generative, their well-being can increase [12]. Some studies have shown a positive association with life satisfaction and well-being [52,53,54], which allows us to understand the satisfaction factor as a key element of the scale of generativity under construction.

Factor three, called “volunteering”, grouped items associated with the performance of formal unpaid activities in the community framework for health care and well-being in general. This coincides with the expression of the style of communal or binding generativity, proposed by Triadó (2018), who maintains a high involvement in caring for others, to the point that even personal wishes and goals become secondary [54].

It is known that the sense of belonging to the community is a predictor of involvement in volunteering activities and that understanding the contributions that older people make to their communities allows us to recognize the concept of civic participation, which includes two different components: (i) social participation, referring to activities that connect people with each other and who are related to caring for others and (ii) community development, which includes most volunteering activities, and political participation, referring to activities that have a clear political intention and that seek to influence the processes of decision-making at any of the levels at which they occur [55].

Thus, volunteering has been associated with numerous indicators of well-being in longitudinal studies, among them: reduction in mortality, increase in physical functionality, improvement in self-evaluation of health, reduction in depressive symptoms, and increase in satisfaction with life [56]. It is also important to highlight the support provided by some environmental factors such as the availability of service roles (volunteering) promoted by an adequate social policy, and institutional efforts to empower the elderly in order to overcome barriers such as poverty, age segregation, and isolation.

The fourth factor, “support networks”, included items on coexistence, friendship, and group status. Generativity integrates personal development with social development and combines an interest in the well-being of future generations with the benefit of finding greater meaning in life and living it more satisfactorily [5]. It is known that involvement in the community can be a source of new development opportunities in old age, offering the elderly significant new roles that compensate for the loss of the role of salaried worker and, at the same time, help to remove traditional images of old age associated with passivity and dependence.

Social networks within the framework of generativity constitute a social platform on which generative interests are developed, so that, according to Arias and Iglesias (2015), they offer opportunities for this adaptation mechanism to be efficient and ultimately have an impact on wellness and health. Generativity is a source of desirable states, which are intrinsically social and extend beyond the generative person and the final states of one’s own health or strictly personal development. These authors point out that it is not the social structure itself that affects the health of the individual but the centrality of the social networks of which it is a part [15].

The relationship that the generative person establishes with other generations is important, whether it is through the care of grandchildren, as caregivers of the sick or fragile people, in civic participation, or in volunteering. Because in these exchanges, higher levels of maturity, psychological and personal growth can be developed, which enables those who are generative to leave their closest environment and learn new skills, expanding their social network and developing a new purpose in life [6,28].

The fifth and last factor, “social support offered”, grouped items on the help offered to others, offering health advice, and the possibility of caring for other people. The reason why the maintenance of physical and cognitive capacities can allow the generative person to use strategies for their own care, learn new strategies, and/or share what they have learned with others [7].

In the case of generativity, care is that associated quality; it is one of the typical areas of generative behavior. Although caring can be a source of stress, some authors such as Netto et al. (2005) highlight that caregivers can also experience endless gains linked to feelings of achievement and personal competence, self-realization, and changes in the philosophy of life that are clearly related to generativity [57].

Two dimensions of generativity can be recognized. One has to do with care-caring for grandchildren, caring for people in a situation of dependency, and the transmission of knowledge through mentoring and participation in intergenerational programs [3], in those that health care represents a key element during the aging process.

Regarding the factors of a scale, the identification of the relevant factors that make it up must be congruent with the construct and the theoretical framework [58,59]. In short, the GeReH was designed through a process of analysis of the theory of psychosocial development, health promotion, and social support networks, where the construct of generativity related to health allowed the design of the GeReH with psychometric properties. However, a continuous review will be necessary to enrich such construct.

The Loyola Generativity Scale has the following dimensions: pass on knowledge or skills to others, mainly other generations, make significant contributions to the betterment of the community or neighborhood, do things that will be remembered for a long time and leave a legacy, be creative or productive, take care and have a responsibility toward other people. Likewise, the Generative Behavior Control List (GBC) includes the dimensions: caring, collaboration, donating, and volunteering [9]; however, they are not outlined to assess satisfaction, mood, and optimism in the activities carried out by the generative people. In this regard, it is important to highlight the importance of social support networks and the social support capital that people in the aging process represent, which is considered in the GeReH.

According to Warburton and Stirling (2007), practicing volunteering would have an impact on health, but at the same time, having good health could explain the beginning and development of voluntary activity, which supports a resource perspective [60]. It is clear that those people who have broader social networks and who trust the rules of reciprocity that derive from them are more likely to be actively involved with their communities, GeReH, pays attention to the above and evaluates volunteering for what considers the promotion of social capital and at the same time can see health-focused generativity as an outcome.

In many urban areas, the social network and the degree of interactions may be weak, although there is a higher demographic density, something that can enhance generativity are institutional changes and also the technological devices that allow it to develop [20], for which the use of technological devices such as “smartphones”, “tablets” and “personal computers” is key [61], for the optimal use of social networks and virtual communication media such as “Facebook”, “WhatsApp”, “E-mail” and “Zoom” among others, with the purpose that generative people can maintain frequent and potentially permanent communication, optimally using technology for telecare during aging, considering a healthy aging approach, especially due to the challenge posed by the COVID-19 pandemic and other emerging situations that may arise. In this context, community generativity programs should not be limited to the face-to-face interaction of participants but should consider the benefits of modern technology.

Regarding self-esteem, this is positively related to generativity in the different factors of the GeReH, probably due to the relationship between self-esteem and success, since the latter is extremely relevant for overcoming life challenges. Lack of self-esteem also produces the reverse of independence, that is, a strong chronic affective dependence, so these people need to cling to someone, constantly bond, and create strong ties. They are people unable to assume their loneliness; in fact, they do not have any authentic relationship, no friends who can offer support. Instead, they demand attention [62].

Generative people find meaning in using their knowledge and skills for their own good; they generally like their work and do it well. Erikson attributes two virtues to the person who has reached the stage of generativity: production (working creatively and productively) and affection (working for the benefit of others) [1].

The transversal axis of the GeReH is self-care for health that allows aging people to take care of themselves, care for others and/or promote health so that it is recognized as a key element in human development during aging. In this context, in congruence with Erikson’s stages and the obvious biological, psychological and social changes that occur in the fifth decade of life [1,14], it is proposed that the application of the instrument developed is around 45 years and over. A person can be independent, autonomous, and generative if he learns new roles; such as promoting community health and participating in social life can be a challenging and rewarding role, with which the aging person can enjoy recognition and visibility in society.

Although the present study did not have a relative objective of differences by gender, it has been observed that the life trajectory of men and women places them with a marked difference in health as their age advances since they reflect the lifestyles they adopted throughout their existence. In this regard, in most cultures, the social role of men is that of the economic provider of the family, exposed to occupational risks and therefore more frequent of some diseases; however, women are longer-lived than men [63].

Differences in the health and lifestyles of aging men and women are largely determined by their social and economic roles. It is expected that these will change as the level of development of the countries advances and as the demographic and epidemiological transitions advance [64].

Gender, as a process of social construction, becomes relevant because it is about the experience of exercising a role socially after being born a man or a woman, it is clear that the individual inevitably develops in a sociocultural setting that coexists with the biological order and that, like this, provides the individual with a series of restrictions, but also opportunities [65].

It is undeniable that information and communication technologies (ICT) can be a tool that contributes to healthy aging, linked to gender, the digital divide refers to the level of crucial digital skills, women on many occasions refer to use and development that is relatively inferior to that of men, as well as a lack of access [66], it is even more profound in the categories of elderly or very old people and constitutes a source of inequality to which attention must also be paid from the perspective of social structure [67]. The lack of knowledge of ICT by many older women results in their difficulty in carrying out certain administrative, communication, or commercial procedures, generating situations of dependency that do not correspond to their abilities and potentialities [68]. The reason why they would have to receive training for its use, which is worth the cost of time and effort if you see the exponential of its use while it can protect health in a pandemic framework such as the one we are facing.

Currently, the use of technologies can and should help to maintain and improve the health of aging people by facilitating communication processes, where distances do not matter, and the use of support networks is made efficient, as well as the support that is provided as well as the one that is provided received, this does not cancel out face-to-face relationships but rather adds the remote route and improves the perception of aging and old age [69].

The GeReH approach qualifies the social support offered related to health, considering taking care of other people and giving health advice, carrying out activities that benefit the community, or contributing to make the world a better one in the framework of volunteering that can include those contributions that the individual makes to the common good, such as helping, teaching and caring, which are determined by their experience, although the scenarios and training for specific generative programs can be created, as can be related to healthy aging.

Caring for grandchildren, young relatives, or other dependent elderly people can allow the elderly to rediscover new generative capacities. In this regard, learning self-care strategies for healthy aging would allow aging people to share knowledge with their peers, thereby achieving social recognition and personal satisfaction [54].

Regarding the possibility of acting as trained informal caregivers, it can have a positive effect in the framework of generativity; it can help to mitigate some of the negative impacts of anxiety related to the negative self-perception of aging (ageism). In fact, interventions based on positive psychology linked to generativity have been implemented with promising success [69].

The GeReH is consistent with studies on the effect of adaptive mechanisms on health, such as those by Fernández-Ballesteros et al. (2010); McAdams et al. (1993); Villar et al. (2013) as well as Arias et al. (2015), who assume that generativity is an adaptive mechanism that contributes to health, particularly point out that being generative has a significant effect on both objective and subjective health, particularly in activities that are relevant for older people to which they guide their adaptive efforts to achieve desired states [6,9,15,70]. Volunteering is also known to lead to increased physical activity, which may represent a plausible behavioral pathway that links generativity to improve well-being [71]. From the relationship between health and volunteering, it is known that, by assuming a productive role as a volunteer, the individual obtains more resources, including larger social networks, as well as more power and prestige, which leads to better mental and physical health [72].

From the perspective of active aging, changes have been generated in the social representation of aging people as well as in the design of devices and environments that favor this type of aging, being prosocial -generative- for social development and for oneself in individual development implies a process of personal importance and the increase in personal skills [73].

Finally, it is important to highlight the relevance of health promotion in community gerontology for healthy aging [74]. Therefore, promoting the training of generative health related to aging people will strengthen the social recognition of this population due to the social impact that its generative function as a promoter of gerontological health would have, and not only that but having trained community groups is extremely important. Measuring generativity related to health with a reliable instrument will make it possible to assess the development of generativity in people who are trained for this purpose.

Among the limitations of the study, it can be highlighted that it is a cross-sectional study, the sample is not representative, empirical results regarding the validity of the instrument are not included since it is the first stage of the design and validity of a reliable instrument. In this regard, the results of a reliable instrument with psychometric properties are presented; however, it is necessary to carry out longitudinal cohort studies and community intervention, considering the training of generative people on the use of current technologies through networks and virtual media so that in turn they train other people in the aging process and promote and strengthen healthy aging.

## 5. Conclusions

The objective of the study was to design a scale to evaluate health-related generativity and analyze its psychometric properties in a population aged 45 years and over in Mexico, considering its internal structure, reliability, and its relationship with self-esteem. In this sense, GeReH is the first scale with a focus on generativity with emphasis on health, multidimensional, culturally contextualized, and reliable for the Mexican population in the aging process. This study allowed the design of the GeReH of 20 items with psychometric properties to evaluate the generativity related to health in people in the aging process (45 years and over), with the possibility of being applied in populations with similar characteristics. Although there are no results of empirical studies regarding the use of the new scale, the importance of considering the use of technological devices is highlighted, for the optimal use of media and social networks, such as “Facebook”, “WhatsApp”, “E-mail”, and “Zoom”, among others.

## Figures and Tables

**Table 1:** Sociodemographic characteristics of the sample (*n* = 450).

Variable	Value
Age, years, mean ± SD	57.5 ± 10.8
Sex (n%)	
Women	296 (65.8)
Men	154 (34.2)
Place of residence (n%)	
Rural	235 (52.2)
Urban	215 (47.8)
Schooling (n%)	
Does not know to read nor to write	29 (6.4)
Can read and write	49 (10.9)
Primary	116 (25.8)
High school	153 (34)
Higher	103 (22.9)
Marital status (n%)	
Single	77 (17.1)
Married	297 (66)
Divorced	38 (8.4)
Widower	38 (8.4)
Community group (n%)	
Religious	79 (17.6)
Political	42 (9.3)
Community	51 (11.3)
Other	44 (9.8)
No group	234 (52)

The inclusion criteria used was that the participants were independent of the BADL and IADL. Exclusion criteria were detection of cognitive impairment and training or work history in health care.

**Table 2:** Factor analysis with the unweighted least-squares extraction method explained variance, reliability index, mean, and standard deviation of the scale items (*n* = 450).

Item	F1	F2	F3	F4	F5
6. I feel good showing interest in others.	0.720				
7. Supporting others is part of my way of being.	0.698				
12. I am satisfied with doing something for others.	0.675				
4. I think about helping other people.	0.577				
47. The way I help others makes me feel good.	0.408				
69. I am satisfied with what I do on a daily basis.		0.648			
20. I am satisfied with what I am doing in my aging process.		0.644			
21. I do many things with humor.		0.511			
52. I try to see the positive in the situations that may arise.		0.485			
40. I meet the goals I set for myself.		0.459			
62. I carry out activities that benefit my community.			0.702		
51. The activities I carry out benefit society.			0.699		
43. I trust that with my contribution the world will be better.			0.484		
34. I would like to do activities for the welfare of my communnity.			0.459		
26. I live with other people besides my family.				0.718	
85. I have friends I can trust.				0.528	
27. I like to be in a group to share experiences.				0.444	
87. I like to agree with others to take care of them.					0.749
86. I would like to give health advice to others.					0.518
89. The support I give to others requires no planning.					0.481
Number of items	5	5	4	3	3
Variance explained by factor	5.71%	1.1%	0.88%	0.67%	0.53%
McDonald’s omega reliability	0.819	0.737	0.742	0.679	0.686
Half	21	20.8	14.4	11.4	10.1
Standard deviation	3.5	3.1	3.4	2.8	2.7

Note: F1 = Generative attitude; F2 = Satisfaction; F3 = Volunteering; F4 = Support networks; F5 = Social support offered.

**Table 3:** Correlation of the factors of the generativity scale (*n* = 450).

	F1 Generative Attitude	F2 Satisfaction	F3 Volunteering	F4 Support Networks	F5 Social Support Offered
F1 Generative attitude	1	0.502 **	0.456 **	0.497 **	0.473 **
F2 Satisfaction		1	0.379 **	0.419 **	0.305 **
F3 Volunteering			1	0.376 **	0.455 **
F4 Support Networks				1	0.377 **
F5 Social support offered					1

** *p* < 0.01.

**Table 4:** Total correlation between the corrected items and Student’s *t*-test. (*n* = 450).

Item	Total Correlation between Elements Corrected	*t* by Student
4. I think about helping other people.	0.578	−14.29, *p* < 0.001
6. I feel good showing interest in others.	0.583	−13.3, *p* < 0.001
7. Supporting others is part of my way of being.	0.538	−11.3, *p* < 0.001
12. I am satisfied to do something for others.	0.525	−10.3, *p* < 0.001
20. I am satisfied with what I am doing in my aging process.	0.391	−7.2, *p* < 0.001
21. I do many things with humor.	0.437	−7.3, *p* < 0.001
26. I live with other people besides my family.	0.499	−10.3, *p* < 0.001
27. I like to be in a group to share experiences.	0.552	−14.1, *p* < 0.001
34. I trust that with my contribution the world will be better.	0.478	−10.8, *p* <0.001
40. I accomplish the goals that I set for myself.	0.472	−9.4, *p* < 0.001
43. I trust that with my contribution the world will be better.	0.475	−11.4, *p* < 0.001
47. The way I help others makes me feel good.	0.592	−12.5, *p* < 0.001
51. The activities I carry out benefit society.	0.488	−11.8, *p* < 0.001
52. I try to see the positive in the situations that may arise.	0.459	−9.2, *p* < 0.001
62. I carry out activities that benefit my community.	0.476	−12.0, *p* < 0.001
69. I am satisfied with what I do on a daily basis.	0.417	−7.2, *p* < 0.001
85. I have friends I can trust.	0.381	−9.2, *p* < 0.001
86. I would like to give health advice to others.	0.567	−14.0, *p* < 0.001
87. I like to agree with others to take care of them.	0.474	−10.5, *p* < 0.001
89. The support I give to others requires no planning.	0.356	−8.0, *p* < 0.001

**Table 5:** Bivariate correlation of generativity factors and high self-esteem.

Generativity Factors	High Self-Steem
F1 Generative attitude	0.270 **
F2 Satisfaction	0.491 **
F3 Volunteering	0.291 **
F4 Support networks	0.194 **
F5 Social support offered	0.161 **

** *p* < 0.01.

## Data Availability

The data set generated and/or analyzed during the study can be requested from the corresponding author.

## Appendix A

**Table A1:** Generativity Relative to Health (GeReH) (90 Item Version) Below is a series of statements about what you feel, think or do, please indicate how often you think you carry out each of them in a range that goes from 1 to 5 considering that: 1 = Never, 2 = Almost never, 3 = Sometimes, 4 = Almost always, and 5 = Always. Your answers are very important, so we ask you to respond truthfully knowing that there are no right or wrong answers and that the information you share is strictly confidential. Please do not leave statements unanswered if you have any questions ask with confidence. Thank you.

	Never	Almost	Sometimes	Almost	Always
		Never		Always	
1. I invite my friends and family to have medical check-ups.					
2. I am pleased that people recognize what I do.					
3. I contribute to the improvement of the environment (I sweep the street, water plants, recycle materials, etc.).					
4. I think about helping other people.					
5. I devote part of my time to taking care of others (children, grandchildren, friends, etc.).					
6. I feel good showing interest in others.					
7. Supporting others is part of my way of being.					
8. My hours of sleep are enough to feel good during the day.					
9. I make plans that include other people.					
10. I feel good when I have shared goals with other people.					
11. I know that meeting my needs is important.					
12. I am satisfied with doing something for others.					
13. I feel like I include myself in other people’s lives when I help them.					
14. I consider myself strong enough to do the common good.					
15. I feel that I am a responsible person when I share with others what has worked for me.					
16. My health status allows me to help others.					
17. I am willing to transmit to others the benefits of taking care of their health.					
18. I donate to those in need.					
19. I would always like to be learning regardless my age.					
20. I am satisfied with what I do in my aging process.					
21. I do many things with humor.					
22. I am interested in being a health promoter in my community.					
23. I take care of sick or dependent people.					
24. When I start something I DO NOT leave it until I finish it.					
25. People DON’T value me the way they used to					
26. I live with other people besides my family.					
27. I like to be in a group to share experiences.					
28. I identify myself with people who see aging as something positive.					
29. I give advice to others whenever they ask for it.					
30I would like to share my experiences with young people.					
31. I am someone who teaches others about my experiences.					
32. I DO NOT always finish the plans that I start.					
33. I intend to pass my knowledge on to others.					
34. I would like to do activities for community welfare.					
35. When I get sick I am able to take care of myself.					
36. I talk about my experiences to improve the well-being of others.					
37. I am happy to help others maintain their health.					
38. I am fully committed to protect the health of others.					
39. I have become invisible to others.					
40. I meet the goals I set for myself.					
41. I am a better person by promoting the health of others.					
42. I would like to attend talks to learn about health issues.					
43. I trust that with my contribution the world will be better.					
44. I want to do something new.					
45. With my experience I help others when they need it.					
46. My life is enriched when I help others.					
47. The way I help others makes me feel good.					
48. I give to others and I receive from them.					
49. It is very important to me to teach that health is a treasure.					
50. In the last twelve months, I have exercise at least three times a week.					
51. The activities I carry out benetit society.					
52. I try to see the positive in the situations that may arise.					
53. The people I help appreciate me.					
54. I am a better human being when I help other people.					
55. I promote health care for other people.					
56. I believe that aging is a time of stagnation.					
57. I am committed mysef to caring for all those around me					
58. I visit sick people to cheer them up.					
59. I identify myself as someone collaborative.					
60. I depend on other people for my care.					
61. When I offer help I am rejected because of old age.					
62. I carry out activities that benefit my community.					
63. I participate in support groups for adults like me.					
64. I feel like my life is heading in a positive direction.					
65. Close to where I live there are places (Health Care Centers or organized groups) where I can volunteer					
66. I would like values such as respect and solidarity to be maintained in order to make the world a better place					
67. I would like to share with others what has worked well for me for my health care.					
68. I feel good when what I do leads me to achieve my goals.					
69. I am satisfied with what I do on a daily basis.					
70. I focus my activities on serving others.					
71. I am self-sufficient in my daily decisions.					
72. I tend to eat my foods low in fat, salt, and sugar.					
73. At my age I can continue learning.					
74. Attending health promotion talks can benefit me.					
75. When someone needs it, I do healing, take blood pressure, give injections, etc.					
76. The people I live with tell me that I inspire trust.					
77. I am concerned about maintaining good health.					
78. I plan things because I know I have time to finish them.					
79. I get a medical checkup at least once a year.					
80. It makes me happy to think of helping someone.					
81. I am delighted to participate in group recreational activities.					
82. I know how to help others.					
83. It satisfies me to have shared goals.					
84. A healthy person is a whole person.					
85. I have friends I can trust.					
86. I would like to give health advice to others.					
87. I like to agree with others to take care of them.					
88. I am happy to commit myself to others.					
89. The support I give to others requires no planning.					
90. I know how to find information to meet my needs.					

## Appendix B

**Table A2:** Generatividad relativa a la salud (GeReH) (Spanish version). A continuación, hay una serie de afirmaciones sobre lo que siente, piensa o hace, por favor indique cuánto tiempo considera que lleva a cabo cada una de ellas en un rango que va del 1 al 5 considerando que: 1 = Nunca, 2 = Casi nunca, 3 = A veces, 4 = Casi siempre y 5 = Siempre. Sus respuestas son muy importantes por lo que le pedimos que responda con total sinceridad sabiendo que no hay respuestas correctas o incorrectas y que la información que comparte es estrictamente confidencial. Por favor, no deje afirmaciones sin respuesta, si tiene alguna pregunta, pregunte con confianza. Gracias.

	Nunca	Casi Nunca	A Veces	Casi Siempre	Siempre
1 Pienso en ayudar a otras personas.					
2 Me siento bien mostrando interés por los demás.					
3 Apoyar a otros es parte de mi forma de ser.					
4 Me satisface hacer algo por los demás.					
5 Estoy satisfecho (a) con lo que hago en mi proceso de envejecimiento.					
6 Muchas cosas las realizo con humor.					
7 Convivo con otras personas además de mi familia.					
8 Me gusta estar en grupo para compartir experiencias.					
9 Me gustaría hacer actividades para el bienestar de mi comunidad.					
10 Cumplo las metas que me propongo.					
11 Confío que, con mi contribución el mundo sea mejor.					
12 La manera en que ayudo a otros me hace sentir bien.					
13 Las actividades que realizo tienen un beneficio para la sociedad.					
14 Procuro ver lo positivo de las situaciones que se me presentan.					
15 Realizo actividades que benefician a mi comunidad.					
16 Estoy satisfecho (a) con lo que hago diariamente.					
17 Tengo amigos (as) en quienes puedo confiar.					
18 Me gustaría dar consejos de salud a otros.					
19 Me gusta ponerme de acuerdo con otros para cuidarlos.					
20 El apoyo que le doy a los demás no requiere planeación.					

## Appendix C

**Table A3:** Generativity Relative to Health (GeReH) (English version). Below, is a series of statements about what you feel, think or do, please indicate how often you think you carry out each one of them in a range that goes from 1 to 5 considering that: 1 = Never, 2 = Almost never, 3 = Sometimes, 4 = Almost always, and 5 = Always. Your answers are very important, so we ask you to respond truthfully knowing that there are no right or wrong answers and that the information you share is strictly confidential. Please do not leave statements unanswered. If you have any questions, ask with confidence. Thank you.

		Never	Almost	Sometimes	Almost	Always
			never		always	
1	I think about helping other people.					
2	I feel good showing interest in others.					
3	Supporting others is part of my way of being.					
4	I am pleased with doing something for others					
5	I am satisfied with what I do in my aging process.					
6	I do many things with humor.					
7	I spend time with other people besides my family					
8	I like to be in a group to share experiences.					
9	I would like to do activities for my community welfare.					
10	I meet the goals I set for myself.					
11	I trust that with my contribution the world will be better.					
12	The way I help others makes me feel good.					
13	The activities I carry out have a benefit for society.					
14	I try to see the positive in the situations that may arise.					
15	I carry out activities that benefit my community.					
16	I am satisfied with what I do on a daily basis.					
17	I have friends I can trust.					
18	I would like to give health advice to others.					
19	I like to agree with others to take care of them.					
20	The support I give to others requires no planning.					
